# Rhythms and prognosis of patients with cardiac arrest, emphasis on pseudo-pulseless electrical activity: another reason to use ultrasound in emergency rooms in Colombia

**DOI:** 10.1186/s12245-020-00319-4

**Published:** 2020-12-04

**Authors:** German Devia Jaramillo, Norberto Navarrete Aldana, Zaira Rojas Ortiz

**Affiliations:** 1 Department of Emergency Medicine, Universidad del Rosario, Bogotá, Colombia; 2 Hospital Universitario Mayor Méderi, Bogotá, Colombia

**Keywords:** Ultrasound, Cardiac arrest, Emergency room, Critical care, Colombia

## Abstract

**Background:**

The cardiac arrest is still an emergency with a bad prognosis. The growing adoption of bedside ultrasound allowed to classify PEA in two groups: the true PEA and the pseudo-PEA. pPEA is used to describe a patient who has a supposed PEA in the absence of pulse, with evidence of some cardiac activity on the bedside ultrasound.

**Objective:**

This work aims to assess the bedside ultrasound use as a predictor for ROSC and survival at discharge in cardiac arrest patients and compare the pseudo-pulseless electrical activity to other cardiac arrest rhythms, including shockable rhythms.

**Materials and methods:**

This is an observational, historic cohort study carried out in the emergency room of the University Hospital Mayor Méderi. Data were collected from all the adult patients treated for cardiac arrest from June 2018 to 2019. An ultrasound was performed to every cardiac arrest patient.

**Results:**

Of a total of 108 patients, the median of the age was 71 years, 65.8% were male subjects, and the most frequent cause for cardiac arrest was the cardiogenic shock (32.4%). ROSC was observed in 41 cases (37.9%) and survival at discharge was 18 cases (16.7%). VF/VT and pPEA were the two rhythms that showed the highest ROSC and survival at discharge. For the pPEA group, we were able to conclude that the cardiac activity type is related to ROSC.

**Conclusion:**

There is a significant difference for ROSC and survival at discharge prognosis among the cardiac arrest rhythms, with better outcomes for VF/VT and pPEA. Among patients with PEA, a routine ultrasound assessment is recommended. The type of cardiac activity recorded during the ultrasound of the cardiac arrest patient might be related to the ROSC and survival at discharge prognosis.

## Background

Despite recent progress on cardiopulmonary resuscitation, cardiac arrest is still an emergency with a bad prognosis, with reports of low survival at discharge rates ranging from 8.3% [1] to 10% [2]. Regarding the cardiac arrest rhythm, shockable rhythms have been reported to have a better survival prognosis in comparison to non-shockable rhythms. Some reports show survival rates of up to 40% with ventricular fibrillation rhythms in opposition to 2.4% with non-shockable rhythms, such as pulseless electrical activity (PEA) [3]. The growing adoption of bedside ultrasound allowed to classify PEA in two groups: the true pulseless electrical activity (PEA) and the pseudo-pulseless electrical activity (pPEA). pPEA is used to describe a patient who has a supposed PEA in the absence of pulse, with evidence of some cardiac activity on the bedside ultrasound [4]. Some reports have shown that pPEA has a better prognosis than true PEA with regard to return of spontaneous circulation (ROSC) and higher survival rates [5, 6]. Hence, this work aims to assess the bedside ultrasound use as a predictor for ROSC and survival at discharge in cardiac arrest patients and compare the pseudo-pulseless electrical activity to other cardiac arrest rhythms, including shockable rhythms such as pulseless ventricular tachycardia and ventricular fibrillation.

## Materials and methods

### Design and study location

Observational, historic cohort study carried out in the emergency room of the University Hospital Mayor Méderi. The emergency room of this high complexity university hospital treats 283,000 adults per year in 110 observation beds, among which 15 beds are located in the adult resuscitation room. The Mayor Hospital is a single, highly complex institution that receives patients from other less complex hospital centers.

### Data source and patients

Data were collected from all the adult patients (≥ 18 years old) treated for cardiac arrest in the emergency room from June 2018 to June 2019. Patients with traumatic cardiac arrest or patients that were referred to other institutions through administrative request were excluded from the study since the follow-up was not possible to accomplish.

### Clinical treatment

According to the protocol, an ultrasound was performed to every cardiac arrest patient during cardiopulmonary resuscitation, except for patients with ventricular fibrillation/ventricular tachycardia (VF/VT) rhythm. This procedure was completed by the specialized medical staff from the emergency room, by using the real-time ultrasound device SonoSite M-Turbo P08792/P09823. Real-time, 2D heart images were obtained with a sector transducer on the parasternal long axis, apical four- or five-chamber view, or subxiphoid view. Subsequently, cardiac activity and its type was assessed and recorded.

### Variables

Collected data includes demographic data (age, sex), pathology type, diagnosis at admission, and functional status before the cardiac arrest. Additional information includes aspects related to cardiopulmonary resuscitation, such as the place of cardiac arrest (pre-admission vs. post-admission), if the resuscitation was witnessed, cardiac arrest time, and cardiac arrest rhythm (PEA, asystole, VF/VT, pPEA). The latter parameter was determined by the activity type: ventricular and valvular cardiac activity (CVA) or only valvular activity (VA). Survival data: ROSC, 24-h survival, and survival at discharge.

### Sample size and statistical analysis

No sampling activities were carried out; data was obtained from all the cardiac arrest patients (patient population census). Continuous variables (age, cardiac arrest time) are presented as the median and interquartile range, after testing normality by Shapiro-Wilk test. Categorical variables are summarized as percentages. Continuous variables were compared by using Wilcoxon’s rank-sum test. Categorical variable comparisons were performed with Pearson’s chi-square test or Fisher’s exact test. All the analyses were carried out on the STATA v.15 (Stata corp) software. *P* values lower than 0.05 were considered as statistically significant.

### Ethical aspects

All patients who were admitted to the emergency room signed a generic form (patient consent F-CME - 22 V.0). They accepted and gave us their written informed consent for the use and publication of their medical records for academic and research purposes. In case the patient is not able to sign, his closest family member signs the authorization document for the handling of his data (form F-CME - 22 V.0). Additionally, this research is considered a without-risk research by Colombian laws (Resolution 8430/1993).

The study design was reviewed and approved by the Hospital Research Committee.

## Results

During the study period, data was collected from 108 patients. The median of the age was 71 years, 65.8% were male subjects, the most frequent place for cardiac arrest was in-patient setting (87.9%) and most of the arrests were witnessed (85.1%). According to family, accompanying persons or previous medical assessments, 97.2% of patients were considered functional for daily-live activities before the cardiac arrest. Ultrasound acquisition was carried out during the cardiac arrest for 81.5% of patients. The most frequent cause for cardiac arrest was the cardiogenic shock (32.4%). Other causes are summarized in Table 1. Median for cardiac arrest time reached 14 min (Table 1).

**Table 1:** Population characteristics (108 patients)

Age	71 years (IQR: 59-77)
Sex	74 (68.5%) men
Place of cardiac arrest	95 (87.9%) in-hospital
Cardiac arrest witnessed	92 (85.1%)
Adequate prior functional status	105 (97.2%)
Cardiac arrest time^a^	14 min (IQR: 6-20 min)
Cause of cardiac arrest	Septic shock 24 (22.2%)
	Hypovolemic shock 10 (9.2%)
	Neurogenic shock 7 (6.4%)
	Cardiogenic shock 35 (32.4%)
	Ventilatory failure 28 (25.9%)
	Unknown 4 (3.7%)
Use of ultrasound	92 (85.1%)

^a^Median (interquartile range)

None of the following variables were found to be significantly correlated to survival at discharge: age (*p* = 0.889), sex (*p* = 0.195), prior functional status (*p* = 1.0), witnessed arrest (*p* = 0.728), or place of cardiac arrest (*p* = 0.451). However, cardiac arrest time correlates to ROSC and survival at discharge (*p* < 0.01) (Table 2).

**Table 2:** Relationship of variables with ROSC and survival at discharge

	Survival at discharge		
Variable	Survive	Dead	***P***
Age^&^	68 (59-79)	71 (59-77)	0.888
Sex			
Male	10 (13.5%)	64 (86.5%)	0.195
Female	8 (23.5%)	26 (76.5%)	
Cardiac arrest time^a^	6 min (4-12)	15 min (10-21)	< 0.001
Place of cardiac arrest			
In-hospital	15 (15.8%)	80 (84.2%)	0.451
Out-of hospital	3 (23.1%)	10 (76.9%)	
Cardiac arrest witnessed			
Yes	15 (16.3%)	77 (83.7%)	0.728
No	3 (18.7%)	13 (81.2%)	
Prior functional status			
Functional	18 (17.1%)	87 (82.9%)	1.0
No functional	0 (0.0%)	3 (100%)	

^a^Median (interquartile range)

The most common cardiac arrest rhythm in the study group was asystole (33.3%), and the least frequent was ventricular fibrillation/ventricular tachycardia (VF/VT) (Table 3). ROSC was observed in 41 cases (37.9 %) and survival at discharge was 18 cases (16.7%). Independency test results were significant when the arrest rhythms were tested against each survival variable (*p* < 0.01). VF/VT and pPEA were the two rhythms that showed the highest ROSC and survival at discharge (Table 3).

**Table 3:** Rhythm during cardiac arrest and outcomes

Rhythm*	*N* (%)	ROSC	24-h survival	Survival at hospital discharge
PEA	23 (21.3%)	2 (8.7%)	1 (4.3%)	0 (0%)
pPEA	33 (30.5%)	20 (60.6%)	16 (48.4%)	11 (33.3%)
ASYSTOLE	36 (33.3%)	10 (27.7%)	6 (16.6%)	1 (2.7%)
VF/VT	16 (14.8%)	9 (56.2%)	9 (56.2%)	6 (37.5%)

*PEA* pulseless electrical activity, *pPEA* pseudo-pulseless electrical activity, *VF/VT* ventricular fibrillation/ventricular tachycardia

*p = <0.001

**Fig. 1:**
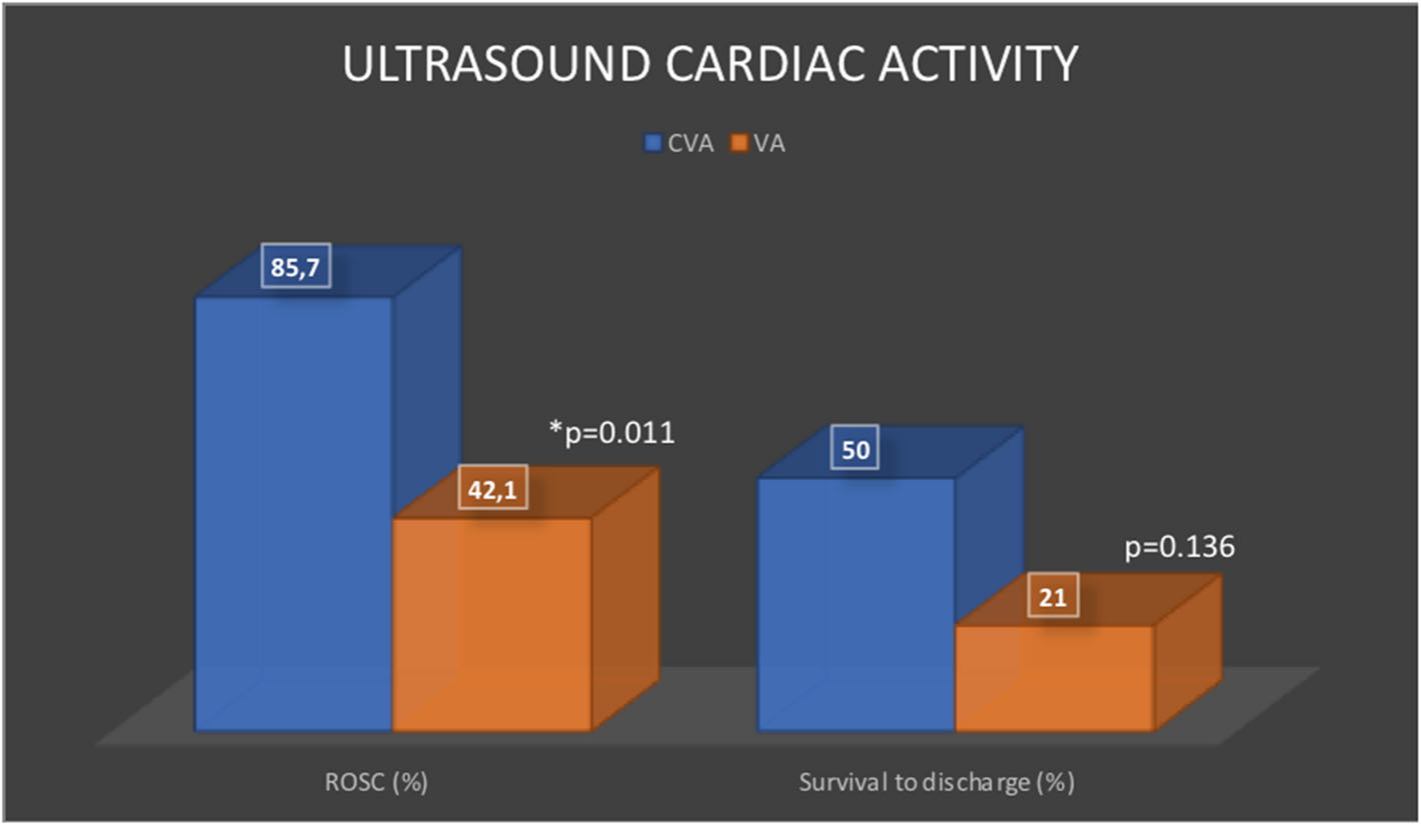
Ultrasound cardiac activity. ROSC, return of sustained circulation; CVA, cardiac and valvular activity; VA, valvular activity only

Finally, especially for the pPEA group, we were able to conclude that the cardiac activity type is related to ROSC, but it is not related to survival at discharge (Fig. 1).

## Discussion

Cardiac arrest is one of the most critical emergencies inside an emergency room. Despite the progress on treatment tools, it is still one of the emergencies with the lowest survival rates after treatment. Therefore, ongoing research for alternative therapies look for improving patient output or identifying subjects that would not receive the benefits of the therapeutic approaches due to a slight or null probability of response. Ultrasound use among non-radiologist physicians has been steadily growing and it becomes more useful for either diagnosis or therapies [7]. In Colombia, the use of ultrasound by non-radiologist physicians, and especially by emergency room medical staff, is scarce due to the limited resources available and the lack of training. Not all the high complexity hospitals have an ultrasound device in the emergency room, even if there is a growing evidence of the helpfulness on the bedside patient setting [8]. In Colombia, we need to increase the body of evidence of benefits from ultrasound use in order to expand the adoption of this tool by well-trained staff. This work was carried out on a cohort of cardiac arrest patients to determine the prognosis of different arrest rhythms, especially on patients with pPEA, which can only be diagnosed by a bedside ultrasound device.

This work showed that sex and age at the cardiac arrest of the studied patients are similar to other studies [9], which render this group comparable to other cohorts in published literature. Global survival at discharge of the studied cohort was 16.6%, which is a high percentage when compared to other reports in literature [10]. This high percentage could be attributed to the fact that most of the cardiac arrests were witnessed and they occurred in an inpatient setting. Nevertheless, among the studied patients, no differences were found for patient output according to place of cardiac arrest, if the cardiac arrest was witnessed or not, and the prior functional status, which could be related to the population size limitations. Regarding the factors associated to ROSC, we found that cardiac arrest time is directly related to ROSC: a 6-min (IR: 4-12) resuscitation has a higher probability of response than a 15-min (IR: 10-21) resuscitation. This is in line with previous studies, where patient survival was related to cardiac arrest time [9]. Therefore, it is advisable to reconsider extended cardiopulmonary resuscitations for patients, since this would represent a futile effort.

VF/VT rhythm was the least common. This finding is in agreement with some reports [11] and it is probably related to advances on acute heart disease prevention, which generally results in cardiac arrest with a VF/VT rhythm, while other pathologies generally result in cardiac arrest with other types of rhythms, especially non-shockable rhythms. Regarding the relationship between the cardiac arrest rhythm and prognosis, we found that PEA and asystole, in comparison with pPEA and VF/VT, showed significant differences for ROSC and survival at discharge. This is a relevant finding, since some reports show that shockable rhythms have better survival prognosis than non-shockable counterparts [12], maybe due to the fact that patients with this activity type have some degree of cellular perfusion, as opposite to patients without electrical activity. It is worth to mention that this study did not compare prognosis between VF/VT and pPEA, thus, we cannot conclude anything from this. However, it is clear that asystole patients have a worse prognosis than patients with some degree of electrical activity on the visoscope.

Concerning the patient group with PEA, this work is in line with previous studies [3, 13] that point to an ultrasound assessment for cardiac arrest patients with PEA, since the presence of electrical activity (i.e., pPEA) could indicate higher probabilities of ROSC or even survival for the patient. Our study showed an important difference at survival level between patients with pPEA and PEA—33.3% vs. 0%. These results are similar to other reports [14, 15] that show ROSC percentages of 50% in patients with pPEA, while only 14.1% in patients with PEA. This proves the important value of ultrasounds for cardiac arrest patients, not only to guide interventional procedures [16] but also to determine the prognosis to assess continuity or the eventual suspension of the cardiopulmonary resuscitation.

For patients with pPEA, we can describe the ultrasound activity, in other words, define if the patient with PEA has a predominant ventricular and valvular activity or only an isolated valvular activity. This study showed that the presence of cardiac and valvular activity resulted in significant differences for ROSC, but not for survival at discharge, when compared to isolated valvular activity. This probably suggests that the type of activity recorded during the ultrasound could be useful for the prognosis of the patient with cardiac arrest, so it is not possible to determine if a resuscitation is done without the use of the ultrasound at the bedside, this justifies the use of the Ultrasound as a tool in the assessment of the patient with cardiac arrest. However, further studies with better experimental designs are needed to test this hypothesis.

## Conclusions

There is a significant difference for ROSC and survival at discharge prognosis among the cardiac arrest rhythms, with better outcomes for VF/VT and pPEA. Among patients with PEA, a routine ultrasound assessment is recommended, since cardiac activity presence is related to a better ROSC and survival at discharge prognosis. Finally, the type of cardiac activity recorded during the ultrasound of the cardiac arrest patient might be related to the ROSC and survival at discharge prognosis.

## Limitations

This study has the limitations of a retrospective study, where some data could be missing. However, this study only included patients with complete and verifiable data from clinical histories. This study was carried out in a single hospital, which might limit the applicability of results. Nevertheless, this study is useful to create a work group to address this topic in Colombia. Finally, unfortunately, the percentages of cardiac arrests in outpatient and inpatient settings were not similar, but this was merely due to it was a study based on daily clinical practice.

## Data Availability

The datasets generated and/or analyzed during the current study are not publicly available due to institutional privacy but are available from the corresponding author on reasonable request.
